# Insights into the control and consequences of breathing adjustments in fishes-from larvae to adults 

**DOI:** 10.3389/fphys.2023.1065573

**Published:** 2023-01-30

**Authors:** Steve F. Perry, Yihang K. Pan, Kathleen M. Gilmour

**Affiliations:** Department of Biology, University of Ottawa, Ottawa, ON, Canada

**Keywords:** gill, ventilation, chemoreceptor, zebrafish, *Danio rerio*, ontogeny

## Abstract

Adjustments of ventilation in fishes to regulate the volume of water flowing over the gills are critically important responses to match branchial gas transfer with metabolic needs and to defend homeostasis during environmental fluctuations in O_2_ and/or CO_2_ levels. In this focused review, we discuss the control and consequences of ventilatory adjustments in fish, briefly summarizing ventilatory responses to hypoxia and hypercapnia before describing the current state of knowledge of the chemoreceptor cells and molecular mechanisms involved in sensing O_2_ and CO_2_. We emphasize, where possible, insights gained from studies on early developmental stages. In particular, zebrafish (*Danio rerio*) larvae have emerged as an important model for investigating the molecular mechanisms of O_2_ and CO_2_ chemosensing as well as the central integration of chemosensory information. Their value stems, in part, from their amenability to genetic manipulation, which enables the creation of loss-of-function mutants, optogenetic manipulation, and the production of transgenic fish with specific genes linked to fluorescent reporters or biosensors.

## 1 Introduction

Arguably, the most thorough review of the control of breathing in fishes was published in 1986 in the *Handbook of Physiology* (Shelton et al., 1986) as part of an exhaustive tome that covered the regulation of ventilation in all ectothermic vertebrates. In the ensuing years, numerous reviews synthesizing aspects of the control of breathing in fishes have appeared (Burleson et al., 1992; Glass, 1992; Gilmour, 2001; Burleson and Milsom, 2003; Gilmour and Perry, 2007; Sundin et al., 2007; Perry et al., 2009a; Perry et al., 2009b; Perry and Gilmour, 2010; Perry, 2011; Perry and Abdallah, 2012; Jonz et al., 2015a; Perry et al., 2016; Perry and Tzaneva, 2016; Tresguerres et al., 2019; Pan and Perry, 2020), including a recent sequel to the 1986 *Handbook* classic (Milsom et al., 2022). The topics covered in these reviews span all levels of respiratory control from chemoreception of the respiratory gases (O_2_, CO_2_ and ammonia) and central integration of afferent sensory input to efferent motor output to the muscles controlling water- or air-breathing. Additionally, several of these reviews include sections on the physiological significance of ventilatory adjustments associated with environmental change or increased physical activity (Perry et al., 2009b; Perry, 2011; Milsom et al., 2022). Thus, one might reasonably question the need for yet another review of piscine respiratory control. Frankly, there is probably no need for another **comprehensive** synthesis. However, in light of recent findings, particularly pertaining to early developmental stages, several emerging themes merit discussion in a brief and focused review. These themes include the ontogeny of chemoreception and the associated respiratory reflexes as well as the molecular and cellular mechanisms underlying the sensing of the two well-studied respiratory gases, O_2_ and CO_2_.

The current review will focus exclusively on ventilation in water-breathing fishes and highlight research on zebrafish (*Danio rerio*) with a specific focus on the early stages of development. The emphasis on zebrafish reflects recent advances in transgenic and loss-of-function (e.g., knockdown, knockout) approaches (Zimmer et al., 2019) that were developed to study the control of breathing in this model species. The review begins with a brief discussion of the ventilatory responses associated with environmental changes in O_2_ and CO_2_, which is followed by more detailed descriptions of the mechanisms underlying the sensing of these respiratory gases. In a final section, we propose areas for future studies that would use genetic techniques amenable to larval zebrafish.

## 2 Ventilatory adjustments to environmental changes in respiratory gases

The volume of water ventilating the gills is a key determinant of O_2_ consumption, hence aerobic metabolic rate. Thus, adjustments to ventilation (in concert with cardiac changes) are used to match O_2_ consumption with metabolic needs. To modify O_2_ uptake rates independently of metabolic rate, ventilation volumes also are controlled by environmental cues linked to modulation of chemoreceptors. Although less well-studied, the excretion rates of CO_2_ and NH_3_ (water-breathers) also are governed, in part, by ventilation volumes. Over a relatively narrow range of ventilation volumes (∼60–150 mL min^−1^), Iwama et al. (1987) reported a significant correlation between O_2_ uptake and CO_2_ excretion yielding a slope of 1.0. The effects of hyperventilation on branchial ammonia excretion are complex and vary according to the prevailing diffusion limitations (Randall and Ip, 2006). Indeed, under so-called resting conditions, the rate of ammonia excretion was unaffected by water flow (Eom et al., 2020). However, under conditions of reduced diffusion limitations (e.g. in fish experiencing elevated plasma ammonia levels and increased expression of Rh ammonia channels), there was a clear correlation between ventilation and ammonia excretion. Owing to the limited data available on mechanisms of ammonia sensing, the following discussion focuses on the ventilatory effects of changes in environmental O_2_ and CO_2_ levels. However, readers interested in the ventilatory effects of the third respiratory gas, NH_3_, are directed to the following publications (Zhang and Wood, 2009; Zhang et al., 2011; Zhang et al., 2013; De Boeck and Wood, 2015; Zhang et al., 2015; Eom et al., 2019; Eom et al., 2020; Eom and Wood, 2021; Porteus et al., 2021).

### 2.1 Environmental O_2_ levels

Numerous aquatic habitats vary spatially and temporally in levels of dissolved O_2,_ ranging from hypoxia [a reduction in O_2_ partial pressure (PO_2_) below that of air-equilibrated water] to hyperoxia (an increase in PO_2_). Although such gradients in ambient PO_2_ have existed through “geological time” (Diaz, 2001), their severity and zones of occurrence are increasing steadily owing to human activities. In response to hyperoxia, fish lower ventilation (hypoventilate) (Wood and Jackson, 1980; Heisler et al., 1988; Kinkead and Perry, 1991; Reid et al., 2005; Vulesevic et al., 2006; Tzaneva and Perry, 2014; Porteus et al., 2015; Porteus et al., 2021). Assuming that the cost of gill ventilation is a significant component of the overall energy budget (Jones and Schwarzfeld, 1974; Steffensen and Lomholt, 1983), hyperoxic hypoventilation may confer some energetic savings. The associated reduction in gas transfer efficiency is of no consequence to blood O_2_ transport because the arterial PO_2_ will remain well in excess of that needed for full saturation of haemoglobin. However, the retention of CO_2_ associated with hypoventilation elicits respiratory acidosis (a lowering of pH caused by an increase in PCO_2_) (Wood and Jackson, 1980), an obvious detrimental consequence of hyperoxia exposure that could impact fish health in commercial aquaculture settings that add supplemental O_2_ to the water (Zimmer and Perry, 2022).

The effects of hypoxia on ventilation (Randall and Shelton, 1963) have received far more attention than those of hyperoxia (Wood and Jackson, 1980). The reader is referred to several extensive reviews that summarise the ventilatory effects linked to hypoxia (Randall, 1982; Shelton et al., 1986; Burleson et al., 1992; Gilmour, 2001; Perry and Gilmour, 2002; Gilmour and Perry, 2007; Perry et al., 2009b; Milsom, 2012). In brief, fish hyperventilate during hypoxia by increasing respiratory frequency (*f*_V_) and/or amplitude (V_AMP_), depending on species. Collectively, these changes in breathing are termed the hypoxic ventilatory response (HVR). Hyperventilation enhances oxygenation of the blood flowing through the gills and therefore serves to minimize the extent of the reduction in arterial PO_2_ (PaO_2_), and hence haemoglobin O_2_ saturation, that is an inevitable consequence of ambient hypoxia (e.g., Holeton and Randall, 1967). The vast majority of studies that have examined the piscine HVR were performed on adults. In recent years, however, the HVR during early development has received renewed and increasing attention. Studies on zebrafish, in particular, have provided insight into the maturation of the HVR and its developmental plasticity while shedding light on the physiological significance of the HVR in larvae lacking fully developed gills. Given its underdeveloped gills, a high surface-to-volume ratio, thin integument and immature autonomic nervous system, the larva should not be considered a miniature version of the adult although some elements of the HVR are conserved.

### 2.2 Ontogeny of the hypoxic ventilatory response

The scarcity of studies on early life stages may reflect the generally held view that the developing gills of larvae do not contribute significantly to whole body O_2_ uptake until they have developed an adult-like morphology (Rombough, 1988; Rombough, 2007). In zebrafish (Rombough, 2002) and rainbow trout (*Oncorhynchus mykiss*) (Fu et al., 2010), the gills do not assume a predominant role in O_2_ uptake until 14 and 27 days post-hatch, respectively. However, despite the apparently minor role of the gills in O_2_ uptake during early stages of development, zebrafish (Jonz and Nurse, 2005) and rainbow trout (Holeton, 1971) exhibit an obvious HVR as early as 3 and 1 day post fertilization (dpf), respectively. Similar hyperventilatory responses before gill maturation were reported for Arctic char (*Salvelinus alpinus*; McDonald and McMahon, 1977) and gar (*Atractosteus tropicus*; Burggren et al., 2016) larvae, although Atlantic salmon (*Salmo salar*) larvae at 50 dpf do not exhibit a HVR (Polymeropoulos et al., 2014).

Developing fishes ventilate their buccal and opercular cavities with increasing regularity as they mature. Additionally, they possess a finely tuned HVR before the gills are fully formed, a time when the skin is thought to be the dominant site of O_2_ uptake. Thus, the physiological significance of the HVR in larvae prior to gill development is not immediately obvious. One possibility is that this seemingly precocious HVR may be present to ensure that O_2_-sensing pathways are operational by the time the larvae become dependent on branchial gas transfer (Jonz and Nurse, 2005). It is also plausible that O_2_ extraction from the buccal cavity, while not significant during normoxia, assumes an increasingly important role in maintaining routine O_2_ consumption during hypoxia. That is while the gills may not yet exhibit the extensive lamellae that confer large surfaces for gas transfer, the vascularized primordial filaments, lamellar buds and the lining of the buccal cavity, itself, are potential gas transfer surfaces. In support of this idea, the critical PO_2_ (P_crit_; the PO_2_ of the water during progressive hypoxia at which MO_2_ can no longer be maintained) was increased in 7 dpf zebrafish larvae prevented from ventilating their buccal cavity (Pan et al., 2019). Clearly despite the absence of a fully developed gill, the HVR in zebrafish larvae older than 7 dpf benefits O_2_ uptake during hypoxia. It is important to note that the elevated ventilation during hypoxia not only raises water flow through the buccal and opercular cavities, but also increases water flow across cutaneous surfaces. This increase in convection over the skin associated with hyperventilation and increased fin movements (see below) likely increases the trans-cutaneous O_2_ diffusion gradient while reducing physical boundary layers; both effects are expected to increase O_2_ uptake across the skin during hypoxia.

The ventilatory responses of larval fish to hypoxia are temporally complex and influenced by age and the severity of the hypoxia (Turesson et al., 2006; Mandic et al., 2019; Pan et al., 2019). For example, the HVR of larval zebrafish exposed to a PO_2_ of 55 mmHg was transient, lasting only 5 min, at 4 dpf, but persisting for the duration of a 30 min exposure at 10 dpf (Pan et al., 2019). Thus, the HVR might easily go undetected if ventilation was measured only at a limited number of time points after beginning hypoxia. The overall HVR consists of several phases even when the hyperventilation persists. The initial response is a rapid increase in *f*_V_ that typically peaks within 5 min of the onset of hypoxia (Mandic et al., 2019; Pan et al., 2019). The next phase of the HVR consists of a decline in *f*_V_ to normoxic values or to a level that is intermediate between resting and peak *f*_V_. The likelihood of *f*_V_ remaining elevated increases with age and the severity of hypoxia (Mandic et al., 2019; Pan et al., 2019). Similar to adults, the period of increased ventilation in larvae <15 dpf is accompanied by stable O_2_ consumption (Mandic et al., 2020). The limited data on larvae indicate that the increased *f*_V_ begins to decline prior to P_crit_ whereas in adults, the peak HVR is sustained slightly beyond P_crit_ (Mandic et al., 2020). Future studies may benefit from longer exposures (e.g., days to weeks), which would enable comparisons of hypoxic acclimation in larvae with data from adults (Vulesevic et al., 2006).

#### 2.2.1 Synchrony of buccal ventilation and fin movements

In early larval stages, breathing is infrequent and irregular (Holeton, 1971; McDonald and McMahon, 1977; Jonz and Nurse, 2005). For example, breathing movements in zebrafish larvae post hatch are rare under normoxic conditions; the HVR is first observed at 3 dpf but even during hypoxia, the buccal movements are irregular until about 8 dpf (Jonz and Nurse, 2005). Qualitatively similar results were observed for rainbow trout and Arctic char, although the transition from irregular to regular breathing movements occurred over a longer time course, corresponding with their slower rate of development (Holeton, 1971; McDonald and McMahon, 1977). During these early developmental stages, the buccal/opercular movements are synchronized with rapid pectoral fin movements, a behaviour presumed to aid gas transfer across the skin by increasing water flow over the body (Rombough, 1988). The synchrony between gill ventilation and pectoral fin movements during hypoxia in zebrafish larvae was studied by Zimmer et al. (Zimmer et al., 2020). Their data demonstrated that the synchronous movements, which are obvious at 4 dpf, are absent by 21 dpf. It was suggested that the loss of synchrony reflects the transition from reliance on cutaneous gas transfer to reliance on branchial gas transfer. To our knowledge, the sensory mechanisms underlying the fin movements during hypoxia have not been investigated (see Zimmer et al., 2020).

#### 2.2.2 Developmental plasticity

The ventilatory responses of adult fish to hypoxia can be modified by the status of the environment during early development. For example, zebrafish embryos treated with hyperoxic water for 7 days (0–7 dpf) exhibited a blunted HVR when exposed as adults to acute hypoxia (Vulesevic and Perry, 2006). However, there was no effect of subjecting larvae to hypoxia or hypercapnia on the responses of adult fish to hypoxia. The plasticity exhibited by zebrafish exposed to hyperoxia during early development (Vulesevic and Perry, 2006) agrees with the results of studies on mammals showing that exposure to hyperoxia during a critical prenatal period may permanently impair the phrenic response to hypoxia (Ling et al., 1997; Donnelly, 2000; Carroll, 2003). The mechanisms responsible for blunting of the HVR in adult zebrafish subjected to hyperoxia as larvae are unknown but as in mammals, may reflect changes to the O_2_ chemoreceptors or afferent neurons (Donnelly, 2000).

Not only can the HVR be shaped by developmental plasticity (Vulesevic and Perry, 2006), but it is possible that the HVR is modified epigenetically in offspring of parents that were exposed to hypoxia. Notably, zebrafish larvae of one (male) or both parents exposed to hypoxia exhibited significant differences in their hypoxia tolerance compared to offspring of parents that had never experienced hypoxia (Ho and Burggren, 2012; Ragsdale et al., 2022). Although no breathing data were obtained (Ho and Burggren, 2012; Ragsdale et al., 2022), it is tempting to speculate that the transgenerational changes in hypoxia tolerance are related to changes in the HVR. Assessing possible epigenetic effects on the HVR is an area that warrants further attention.

#### 2.2.3 Aquatic surface respiration

In naturally hypoxic aquatic habitats, a thin layer of water at the surface tends to be enriched with O_2_ relative to the bulk water (Burggren, 1982). Fish exploit this microenvironment with a behavioural adaptation termed aquatic surface respiration (ASR) (Kramer and Mehegan, 1981). During ASR, fish rise to the surface and skim the uppermost layer to ventilate their gills with O_2_-enriched water (Gee et al., 1978; Kramer and McClure, 1982). Aquatic surface respiration has been studied almost exclusively in adult fishes (Chapman and McKenzie, 2009) where it has been shown to increase blood oxygenation during severe hypoxia (Burggren, 1982) as well as survival (Kramer and McClure, 1982). To our knowledge, ASR has been examined rarely in juveniles (Weber and Kramer, 1983; Sloman et al., 2008) and in larvae of a single species, zebrafish (Abdallah et al., 2015b; Mandic et al., 2022), in which ASR increased as the severity of hypoxia was increased. In zebrafish, the age of onset of ASR during acute hypoxia is 5 dpf (Abdallah et al., 2015b). At earlier developmental stages, ASR may be constrained by the lack of swimbladder inflation that is needed to control buoyancy (Abdallah et al., 2015b). Similar to adult zebrafish, ASR improves survival during severe hypoxia in larvae older than 5 dpf (Mandic et al., 2022). Indeed, when exposed to a level of hypoxia of 16 mmHg, the survival of larvae (>5 dpf) performing ASR was similar to that of normoxic larvae at close to 100%. Larvae (>5 dpf) spent approximately 30% of their time engaged in ASR, which was similar to the situation in adults experiencing the same level of hypoxia (16 mmHg) (Mandic et al., 2022). The energetic costs associated with extended periods of ASR in zebrafish larvae are unknown but are likely to be low given their state of neutral buoyancy (Lindsey et al., 2010). Conversely, longer time spent at the surface is likely to increase the risk of predation.

### 2.3 Environmental CO_2_ levels

Elevated levels of dissolved CO_2_ in aquatic habitats (interchangeably termed hypercapnia or hypercarbia) arise naturally from biological respiration and microbial decomposition of organic matter, especially in tropical waters with high biomass including the so-called “blackwaters” of the Amazon basin (Furch and Junk, 1997; Rasera et al., 2013). Additionally, eutrophication is a major cause of hypercapnia that is worsening globally owing to pollution, the warming of aquatic environments and increasing levels of atmospheric CO_2_.

As with hypoxia, most studies that assessed the ventilatory effects of hypercapnia were conducted on adult fish. Although PCO_2_ thresholds of onset vary widely, a ubiquitous response to hypercapnia is hyperventilation (Gilmour, 2001; Gilmour and Perry, 2007; Perry et al., 2009a; Milsom, 2012; Perry and Abdallah, 2012; Tresguerres et al., 2019) that, in contrast to hypoxia, typically results from increases in V_AMP_ rather than *f*_V_ (Milsom et al., 2022). The regulation of ventilation by ambient CO_2_ or associated changes in the internal acid-base status, while important, is secondary to the dominant role of O_2_ status in the control of breathing in fishes. In adult zebrafish, episodic breathing is a relatively common occurrence under resting conditions (e.g., 20% of individuals examined) (Vulesevic et al., 2006). Unlike hypoxia, which alters breathing patterns from episodic to continuous, episodic breathing is unchanged by hypercapnia (Vulesevic et al., 2006; Milsom et al., 2022). Currently, there is no explanation for the different patterns of ventilatory responses to hypoxia and hypercapnia, which are presumably to be triggered by the same chemoreceptors (see below).

### 2.4 Ontogeny of the hypercapnic ventilatory response

Little is known about the respiratory reflexes associated with hypercapnia in developing fish. Indeed, the only species that has been studied during larval stages is zebrafish. Unlike adults, which increase ventilation during hypercapnia exclusively by adjusting V_AMP_ (see above), larval zebrafish hyperventilate by markedly increasing *f*_V_ when exposed to elevated ambient CO_2_ (Kunert et al., 2022). To date, there are no reliable methods to measure V_AMP_ in zebrafish larvae; thus, it is conceivable that V_AMP_ increases in concert with changes in *f*_V_. Regardless, it would be useful to determine the time of development when the ventilatory response to hypercapnia shifts from altering *f*_V_ to altering V_AMP_. As in adult fishes, the breathing response of zebrafish larvae to hypercapnia is caused specifically by an increase in ambient PCO_2_ rather than the associated acidification of the water (Kunert et al., 2022). The benefit, if any, of the hypercapnic hyperventilation in larvae, which occurs concurrently with tachycardia (Miller et al., 2014), is unknown.

## 3 Sensory and molecular mechanisms of chemoreception

The reflex ventilatory responses to hypoxia and hypercapnia described above are thought to be triggered by the activation of peripheral chemoreceptors. However, much remains to be learned about the molecular mechanisms of gas sensing as well as the afferent sensory pathways and neural circuitry that process chemosensory signals. As with reflex ventilatory responses, most studies of sensory mechanisms have focused on adult fish or chemosensory cells isolated from adult fish. It is only recently that the power of larval zebrafish as a model for investigating chemoreceptor function *in vivo* has begun to be realized. Below, the evidence that neuroepithelial cells (NECs) serve as O_2_ and CO_2_ chemoreceptors is briefly surveyed and our current understanding of the molecular mechanisms of chemoreception is discussed.

### 3.1 Hypoxia

#### 3.1.1 Peripheral chemoreceptors

The O_2_ chemoreceptors in fishes are believed to be NECs that were first described by Dunel-Erb et al. (1982). In adult fish, they are found on the tips of gill filaments and in larvae, they are located on the skin prior to gill maturation (Jonz and Nurse, 2006; Coccimiglio and Jonz, 2012). NECs also may be present on cutaneous surfaces in adults of some species including the mangrove rivulus (*Kryptolebias marmoratus*) (Regan et al., 2011; Rossi et al., 2020) and giant mudskipper (*Periophthalmodon schlosseri*) (Zaccone et al., 2017).

Gill NECs are characterized by the presence of dense-cored vesicles containing serotonin (5-HT) and their innervation is derived from a plexus of nerve fibres (Dunel-Erb et al., 1982; Bailly et al., 1989; Bailly et al., 1992; Jonz and Nurse, 2003). Initial suggestions of gill NECs being O_2_ chemoreceptors were based on their morphology, location and innervation patterns (Dunel-Erb et al., 1982; Bailly et al., 1992; Jonz and Nurse, 2003; Bailly, 2009). Substantial indirect evidence supports a role for the NECs as peripheral O_2_ chemoreceptors, largely based on their morphological changes (hypertrophy, hyperplasia and increased numbers of neuron-like processes) during exposure to prolonged hypoxia (Jonz et al., 2004; Burleson et al., 2006; Regan et al., 2011; Shakarchi et al., 2013; Rossi et al., 2020; Pan et al., 2021a). Direct evidence that gill NECs are O_2_ sensitive was provided by whole-cell patch-clamp recordings *in vitro* from NECs isolated from adult gill filaments. Specifically, patch clamp experiments demonstrated hypoxia-induced membrane depolarization in isolated gill NECs under current-clamp conditions in zebrafish (Jonz et al., 2004) and channel catfish (*Ictalurus punctatus*) (Burleson et al., 2006). Additional direct evidence was provided by measuring intracellular [Ca^2+^] ([Ca^2+^]_i_) and synaptic vesicle activity in isolated goldfish (*Carassius auratus*) NECs (Zachar et al., 2017).

There is less evidence supporting the notion that skin NECs are O_2_ chemoreceptors. In zebrafish, skin NECs exhibiting innervation are evident in embryos at 1 dpf, before the onset of behavioral responses to hypoxia (Coccimiglio and Jonz, 2012). A population of skin NECs also expresses synaptic vesicle protein, suggesting a capacity for the secretion of neurotransmitters (Coccimiglio and Jonz, 2012). However, all evidence of hypoxia sensitivity in skin NECs is indirect, based on their increasing density and/or size under hypoxic conditions (Regan et al., 2011; Coccimiglio and Jonz, 2012; Rossi et al., 2020), or elimination of the HVR following their partial chemical denervation (Coccimiglio and Jonz, 2012). In contrast, however, NEC development in larval mangrove rivulus is largely unaffected by environmental O_2_ levels (Cochrane et al., 2021). Thus, the current evidence supports hypoxia sensitivity of isolated gill NECs *in vitro*, whereas direct evidence for hypoxia sensitivity in skin NECs is lacking. Additional research, especially *in vivo*, is required to establish both gill and skin NECs as piscine O_2_ chemoreceptors.

#### 3.1.2 Molecular mechanisms and pathways of O_2_ sensing

The sensing of O_2_ begins with the detection of PO_2_ changes by an O_2_ sensor within the chemoreceptor, which leads to the inhibition of K^+^ channels and subsequent membrane depolarization. Cytosolic Ca^2+^ levels increase, through either extracellular Ca^2+^ entry *via* voltage-dependant Ca^2+^ channels or the release of Ca^2+^ from intracellular stores, thereby facilitating vesicle fusion and the release of neurotransmitters. This neurosecretion activates the afferent nerve fibers innervating the chemoreceptors, resulting in the signal being transmitted to the central nervous system (CNS) where it is processed to elicit downstream responses (Gonzalez et al., 2010; Zachar and Jonz, 2012; Prabhakar, 2013; Lopez-Barneo et al., 2016; Ortega-Sáenz et al., 2020).

Regardless of vertebrate class, the molecular mechanisms underlying acute O_2_ sensing remain elusive (Rakoczy and Wyatt, 2018). The earliest ideas for molecular O_2_ sensing were based on mitochondrial inhibition. In this scheme, inhibition of the mitochondrial electron transport chain under hypoxic conditions leads to decreased ATP production that is sensed by ATP-sensitive K^+^ channels, resulting in depolarization and neurotransmitter release (Duchen and Biscoe, 1992a; Duchen and Biscoe, 1992b; Wyatt and Buckler, 2004; Varas et al., 2007). In addition, the decrease in ATP may cause AMP to rise, which could activate AMP-activated protein kinase (AMPK), further phosphorylating membrane ion channels and leading to depolarization; thus, AMPK is a candidate O_2_ sensor (Evans, 2004). Furthermore, disruption of mitochondrial complex I on the electron transport chain could increase production of reactive oxygen species (ROS), changing the redox status of membrane ion channels and thus initiating excitation (Fernandez-Aguera et al., 2015; Gao et al., 2017). The gasotransmitters CO and H_2_S also have been proposed as components of the molecular mechanism for O_2_ sensing (Li et al., 2010; Buckler, 2012; Yuan et al., 2015). Lactate is one of the newest candidates to be proposed as a member of the O_2_ sensing pathway. Chang et al. (2015) demonstrated that the olfactory receptor encoded by *Olfr78* is sensitive to lactate, which may accumulate during hypoxia. *Olfr78* knockout mice did not exhibit a HVR but responded normally to hypercapnia. However, the idea of lactate being involved in O_2_ sensing is not universally accepted because the original results obtained using *Olfr78* knockout mice were not fully reproducible (Chang et al., 2018; Torres-Torrelo et al., 2018). It is likely that additional molecular mechanisms for O_2_ chemoreception will be proposed. Indeed, it is likely that ultimately multiple mechanisms will be identified for O_2_ sensing within the chemoreceptors (Kumar and Bin-Jaliah, 2007).

In fishes, the molecular mechanisms of O_2_ chemoreception have received less attention, and only H_2_S and lactate have been examined in the context of cellular (NEC) O_2_ sensing. In trout branchial tissues where gill NECs are located, genes for the H_2_S-synthesizing enzymes, cystathionine *ß*-synthase and cystathionine γ-lyase, are expressed (Olson et al., 2008). Further, gill homogenates produce H_2_S enzymatically, a process which is inhibited by high levels of O_2_ (Olson et al., 2008). Behaviourally, intrabuccal injection of H_2_S in anaesthetized trout produced increases in *f*_V_ and V_AMP_ similar to those of the HVR (Olson et al., 2008). Similar results were obtained in zebrafish larvae in which H_2_S elicited hyperventilation that was blocked by preventing endogenous H_2_S synthesis throughout the body, including in the cystathionine γ-lyase-containing skin NECs (Porteus et al., 2014). Additionally, H_2_S exposure increased [Ca ^2+^]_i_ in NECs isolated from zebrafish gill and resulted in membrane depolarization similar to that observed under hypoxia (Olson et al., 2008; Perry et al., 2016). Lactate can also elevate [Ca ^2+^]_i_ in isolated killifish (*Fundulus heteroclitus*) NECs (Leonard et al., 2022). In addition, lactate also elevates gill ventilation in a dose-dependent manner independent of pH changes in both the striped catfish (*Pangasianodon hypophthalmus*) and trout, with this response being attenuated following denervation of the first gill arch (Thomsen et al., 2017; Thomsen et al., 2019). Thus the existing evidence suggests that both H_2_S and lactate have the potential to participate in O_2_ sensing within NECs in fish. Two other gasotransmitters, CO and NO, also can modify ventilatory responses in fishes, with CO inhibiting ventilation in goldfish (Tzaneva and Perry, 2014) and zebrafish (Tzaneva and Perry, 2016). Interestingly, the effects of NO on ventilation are not only dependent on developmental age–NO stimulates breathing in zebrafish larvae while inhibiting breathing in adults (Porteus et al., 2015)–but also on HIF-1α, as NO is unable to contribute to the HVR in mutant larvae lacking HIF-1 α. However, there is no direct evidence that CO or NO participates in O_2_ sensing specifically at the level of NECs (Olson et al., 2012; Mandic et al., 2019). A recent single-cell transcriptomic analysis of the zebrafish gill (Pan et al., 2022) revealed high expression of genes encoding for NADH dehydrogenase and cytochrome c oxidase in NECs, genes related to mitochondrial function that have been implicated in O_2_ sensing in mammals.

Under voltage-clamp conditions, isolated gill NECs from adult zebrafish (Jonz et al., 2004) and channel catfish (Burleson et al., 2006) respond with a decrease in K^+^ current that is insensitive to voltage-dependent K^+^ channel blockers but sensitive to the background K^+^ channel blocker, quinidine (Jonz et al., 2004). In addition, hypoxia caused a reversible depolarization that was associated with a conductance decrease in isolated zebrafish gill NECs under current-clamp conditions; this response was reduced in the presence of quinidine (Jonz et al., 2004). In isolated gill NECs from goldfish, hypoxia exposure resulted in increases in [Ca^2+^]_i_ and vesicular activity that were blocked by the Ca^2+^ channel blocker, cadmium, and the L-type Ca^2+^ channel blocker, nifedipine (Zachar et al., 2017). These data suggest that hypoxia sensing in gill NECs is mediated by inhibition of a background K^+^ channel leading to depolarization, an increase in [Ca^2+^]_i,_ and possibly neurosecretion, similar to that observed in glomus cells of the mammalian carotid body (Lopez-Barneo et al., 2016).

Although there is evidence that trout NECs appear “degranulated” after hypoxia exposure (Dunel-Erb et al., 1982), and that NECs possess the synaptic vesicle protein SV2 indicative of neurosecretion potential (Jonz et al., 2004), there is no direct evidence of any neurotransmitter being released by NECs under hypoxic conditions. Serotonin (5-HT) is the only neurotransmitter directly and routinely identified in fish NECs, but there is an array of neurotransmitters that may enable signal transduction between NECs and the afferent neurons, and which have been shown to modulate breathing. The neuroendocrine control of breathing in fish was recently reviewed (Reed and Jonz, 2022) and thus, only a brief summary will be provided here. Using isolated gill arches from rainbow trout, application of acetylcholine resulted in potent stimulation of afferent neural activity, while 5-HT and dopamine caused brief, small bursts in neural activity followed by mild inhibition (Burleson and Milsom, 1995). Adrenaline and noradrenaline did not elicit any neural responses (Burleson and Milsom, 1995). However, when neurotransmitters were administered through intra-vascular injection or applied externally, 5-HT, adrenaline, noradrenaline, acetylcholine and purines generally stimulated ventilation, whereas dopamine inhibited ventilation (Burleson and Milsom, 1995). A similar inhibitory effect of dopamine on ventilation was reported in larval zebrafish (Shakarchi et al., 2013). In addition, single-cell transcriptomic analysis of the zebrafish gill showed that NECs and neurons express genes encoding transmembrane receptors for serotonergic, cholinergic and dopaminergic neurotransmission (Pan et al., 2022). These results are broadly consistent with the mammalian situation in which acetylcholine and purines are excitatory carotid body neurotransmitters whereas dopamine is inhibitory (Leonard et al., 2018). However, unlike in mammals in which neurotransmitter secretion from the carotid body was demonstrated, studies in fish, while establishing a direct link between neurotransmitters and ventilation, have not yet revealed the specific site of action. Future studies are needed to directly examine neurosecretion from NECs under hypoxic conditions.

Upon activation of the chemoreceptors and the presumed release of neurotransmitters, the signal is transmitted to the CNS leading to downstream ventilatory responses. Existing data suggest that cranial nerves IX (glossopharyngeal) and X (vagus) are the afferent neurons innervating the gill chemoreceptors. Thus, total gill denervation of cranial nerves IX and X decreased the magnitude of the HVR in nine species over a wide range of taxonomic diversity (Milsom, 2012) whereas rhythmic stimulation of cranial nerve X entrains the respiratory rhythm in carp (*Cyprinus carpio*) (De Graaf and Roberts, 1991). Using genetically encoded calcium sensors GCaAMP6s under control of the pan-neuronal promoter *elavl3*, Rosales et al. (2019) showed that in zebrafish, the average magnitude of Ca^2+^ transients increased within the sensory ganglia of cranial nerve X upon exposure to hypoxia, providing arguably the first direct *in vivo* evidence for any of the O_2_ sensing components in a fish. Central projections from the ganglia of cranial nerves IX and X enter the hindbrain at presumptive rhombomere 6 and *via* a series of nerve roots to form a ‘plexus’ (Kucenas et al., 2006). Given that the preBötzinger complex, which is the origin of respiratory motor output in mammals, arises from rhombomeres 6 and 7 (Mellen and Thoby-Brisson, 2012), and also that rhombomere 7 is essential for gill and buccal bursts in bullfrog tadpoles (Duchcherer et al., 2013), it is reasonable to hypothesize that O_2_ signals originating in the chemoreceptors are conveyed to the respiratory motor output centres in the hindbrain *via* cranial nerves IX and X.

### 3.2 Hypercapnia

#### 3.2.1 Peripheral chemoreceptors

In water-breathing adult fishes, evidence for a primarily branchial location of CO_2_ chemosensors has come from studies in which CO_2_-stimulated ventilatory responses were eliminated by denervation or extirpation of one or more gill arches (Burleson and Smatresk, 2000; Reid et al., 2000; Sundin et al., 2000; McKendry et al., 2001; Perry and Reid, 2002; Florindo et al., 2004; Bojink et al., 2010). These studies also revealed a predominant role for the first gill arch in eliciting CO_2_-stimulated ventilatory responses in some species [rainbow trout (Perry and Reid, 2002) and jeju *Hoplerythrinus unitaeniatus* (Bojink et al., 2010), but see also (Sundin et al., 2000)], and they identified cranial nerves IX and X as those responsible for transmitting branchial CO_2_ chemoreceptor activity to the brain. In the tambaqui *Colossoma macropomum*, total gill denervation did not completely eliminate ventilatory responses to CO_2_, suggesting that extra-branchial CO_2_ chemoreceptors may be present in some species (Milsom et al., 2002; Florindo et al., 2004). These extra-branchial receptors are likely to be associated with the orobranchial cavity or other peripheral sites because the balance of evidence does not support the presence of central CO_2_/pH chemoreceptors in strictly water-breathing fishes (for discussion, see Tresguerres et al., 2019; Milsom et al., 2022).

The branchial chemoreceptors appear to respond primarily to water CO_2_ rather than to water pH or to changes in blood CO_2_/pH (reviewed by Gilmour and Perry, 2007; Milsom, 2012). Evidence to support this consensus has come from studies that have attempted to independently manipulate water *versus* blood CO_2_ *versus* pH. For example, injection of CO_2_-equilibrated water into the buccal cavity elicits hyperventilation, whereas injection of isocapnic water adjusted to the pH corresponding to that of the CO_2_-equilibrated water has little or no effect (Reid et al., 2000; Sundin et al., 2000; Perry and McKendry, 2001; Gilmour et al., 2005; Bojink et al., 2010). These data argue for the importance of water CO_2_ rather than water pH as the proximate factor controlling ventilation during ambient hypercapnia. A comparison between injection of CO_2_-enriched water into the buccal cavity and injection of CO_2_-enriched saline into the vasculature similarly argues for the importance of water CO_2_, with injection of CO_2_-enriched saline being without effect on ventilation (Perry and McKendry, 2001; Perry and Reid, 2002; Gilmour et al., 2005; Bojink et al., 2010). In addition, treatments that raise internal (but not water) CO_2_ levels do not elicit hyperventilatory responses. For example, arterial CO_2_ tension more than doubled in tambaqui treated with the carbonic anhydrase inhibitor acetazolamide (to inhibit CO_2_ excretion), yet ventilation did not change until the fish were exposed to aquatic hypercapnia (Gilmour et al., 2005). Where questions remain is with the possibility of internally-oriented receptors that detect acid-base status. Although injections of acidified saline into the vasculature (Reid et al., 2000; Sundin et al., 2000; Bojink et al., 2010) or acetazolamide treatment (Gilmour et al., 2005) generally have not evoked ventilatory responses, several studies have reported results that are consistent with ventilation being adjusted by blood acid-base status (for detailed discussion, see Gilmour and Perry, 2007; Tresguerres et al., 2019). In particular, a close correspondence between ventilation and blood acid-base status during recovery from exhaustive exercise was observed in rainbow trout. Treatment of trout with carbonic anhydrase to enhance CO_2_ excretion alleviated not only the acid-base disturbance, but also the accompanying hyperventilation (Wood and Munger, 1994). Thus, the door remains open to the possibility that ventilation may be adjusted according to internal acid-base status, with further research needed to refute or confirm this possibility.

Taken as a whole, the studies of ventilatory responses to hypercapnia suggest that CO_2_ chemoreceptors are located in the gill of (adult) fishes and respond primarily to changes in water CO_2_ levels. As with hypoxia, NECs located in the gill of adult fishes are thought to serve as the CO_2_ chemoreceptors. In comparison with hypoxia, however, very few studies have investigated ventilatory responses to CO_2_ in larvae, and there are no data that directly link cutaneous NECs to CO_2_-stimulated ventilatory responses. Changes in NEC abundance, size and transcriptome in response to changes in environmental O_2_ levels have provided indirect evidence of NEC involvement in O_2_ chemoreception (see above). For CO_2_ chemoreception, however, the data are both sparse and mixed. Acclimation to hypercapnic conditions for 28 d had no impact on the density of branchial (serotonin-positive) NECs in adult zebrafish (Vulesevic et al., 2006). On the other hand, increases in branchial NEC density were reported in mangrove rivulus acclimated to hypercapnia or to acidic water, although whether there were functional consequences of the changes in NEC density was not explored (Robertson et al., 2015). Overall, the paucity of data makes it difficult to draw any firm conclusions. However, there is evidence that branchial NECs detect and respond to hypercapnia in a manner that is consistent with their involvement in CO_2_-evoked reflexes.

Direct evidence that branchial NECs can serve as CO_2_ chemoreceptors was obtained from two studies of cellular responses to CO_2_/pH in NECs isolated from the gills of adult zebrafish. Qin et al. (2010) reported that exposure to hypercapnia caused depolarization in a subset of the NECs that were tested, with the magnitude of the response increasing with increasing partial pressure of CO_2_. The NECs that responded to increases in CO_2_ also depolarized in response to lowering of O_2_ levels, indicating that at least some NECs are dual sensors of O_2_ and CO_2_. Subsequently, an increase in [Ca^2+^]_i_ in response to hypercapnia was reported by Abdallah et al. (2015a). Although both responses were elicited by hypercapnia, the sensory mechanisms involved appear to differ, with a CO_2_-stimulated change in intracellular pH being required for depolarization of the NEC (Qin et al., 2010), but a fall in extracellular pH that is independent of CO_2_ being required for the increase in [Ca^2+^]_i_ (Abdallah et al., 2015a). Further research is needed to resolve this difference, and to reconcile the apparent discrepancy between sensory mechanisms at the cellular level (i.e. where extracellular pH is an adequate stimulus for a change in [Ca^2+^]_i_) and the stimuli that elicit ventilatory responses at the whole-animal level (i.e. changes in water CO_2_ but not pH; see above). In addition, there is a need to link activation of CO_2_-sensing NECs directly to the occurrence of CO_2_-stimulated ventilatory reflexes.

#### 3.2.2 Molecular mechanisms and pathways of CO_2_ sensing

Current understanding of the signalling associated with NEC CO_2_ sensing is based on the two studies mentioned above on NECs isolated from adult zebrafish gills (Qin et al., 2010; Abdallah et al., 2015a), and from studies in which cardiorespiratory responses to CO_2_ in larval zebrafish were investigated (Miller et al., 2014; Koudrina et al., 2020; Kunert et al., 2022). As with O_2_ sensing (Jonz et al., 2004), the initial cellular response to CO_2_ is depolarization, which is caused by the inhibition of K^+^ flux through background K^+^ channels (Qin et al., 2010). It is likely that multiple K^+^ channels are present in NECs, with evidence supporting the involvement of TASK-2 (Koudrina et al., 2020), a member of the tandem-pore domains in a weak inward rectifying K^+^ channel (TWIK)-related acid-sensitive K^+^ (TASK) channel family. Functional studies carried out on zebrafish TASK-2 expressed in HEK-293 cells revealed that it is inhibited by intracellular or extracellular acidification as well as by increases in CO_2_ (Peña-Münzenmayer et al., 2013). Notably, the inhibition of TASK-2 by CO_2_ included both a contribution of intracellular acidification and a direct effect of CO_2_ that was not dependent on changes in intracellular pH. Therefore, the properties of TASK-2 channels are consistent with the requirements for CO_2_ sensing in NECs. Immunohistochemistry was used to demonstrate that TASK-2 is expressed by serotonin-positive NECs in the skin of larvae and the gills of adult zebrafish (Koudrina et al., 2020). A functional role for TASK-2 was identified using antisense oligonucleotide morpholinos to knock down TASK-2 and/or its paralog TASK-2b, which attenuated the hyperventilatory responses to hypercapnia in 4 dpf zebrafish larvae (Koudrina et al., 2020). Interestingly, the genes that encode for TASK-2 (*kcnk5a*) and TASK-2b (*kcnk5b*) were not detected in a single-cell transcriptomic analysis of zebrafish NECs (Pan et al., 2022) (nor was *ca17a*, see below). However, the focus of Pan et al. (2022) was on NECs that respond to hypoxia. If only a subset of NECs serve as bimodal sensors of O_2_ and CO_2_ (Qin et al., 2010), then it could be difficult to detect differential expression of transcripts specific to CO_2_ sensing.

CO_2_-induced depolarization of NECs is expected to cause an increase in [Ca^2+^]_i_. Consistent with this pathway, Abdallah et al. (2015a) documented increases in [Ca^2+^]_i_ in isolated zebrafish NECs exposed to hypercapnia, with Ca^2+^ being derived primarily from intracellular stores. The increase in [Ca^2+^]_i_, in turn, is expected to lead to neurosecretion, transmission of the sensory signal to the brain and activation of the appropriate ventilatory response. However, currently there are no data to support this pathway, nor has any specific neurotransmitter been identified as being secreted. Serotonin is viewed as likely to be involved owing to its status as the predominant neurochemical in NECs (Porteus et al., 2012) and the ability of serotonin receptor agonists to elicit hyperventilatory responses in zebrafish larvae (Jonz et al., 2015b; for reviews see Pan and Perry, 2020; Reed and Jonz, 2022). Although, as alluded to above (section 3.1.2), the fact that certain neurochemicals evoke hyperventilation does not necessarily indicate that they are secreted by NECs.

The CO_2_ sensing mechanism likely involves carbonic anhydrase (CA), specifically the cytosolic isoform Ca17a (Ferreira-Martins et al., 2016). Immunohistochemistry supports the presence of CA or Ca17a specifically in larval cutaneous and adult branchial NECs of zebrafish (Qin et al., 2010; Miller et al., 2014; Kunert et al., 2022). Pharmacological inhibition of CA, as well as specific knockdown or knockout of Ca17a, blunts the cardiorespiratory responses to hypercapnia in zebrafish larvae (Miller et al., 2014; Kunert et al., 2022). These data argue that Ca17a contributes to CO_2_ sensing, yet its specific role at the cellular level remains unclear. Inhibition of CA both slowed the rate and reduced the magnitude of CO_2_-stimulated depolarization in isolated branchial NECs (Qin et al., 2010). In conjunction with the observation that extracellular acidification was not needed to elicit depolarization, these data suggest that depolarization occurs in response to CO_2_ entry into the cell and its CA-catalyzed hydration to H^+^, resulting in intracellular acidification. However, intracellular acidification was not sufficient to elicit a [Ca^2+^]_i_ response, nor did inhibition of CA alter the [Ca^2+^]_i_ response to hypercapnia even though intracellular acidification was slowed (Abdallah et al., 2015a). Indeed, extracellular acidification alone was sufficient to elicit a [Ca^2+^]_i_ response (Abdallah et al., 2015a). Resolving these discrepancies likely will provide insight into the role of Ca17a in CO_2_ sensing.

## 4 Knowledge gaps and perspectives

Despite decades of research focused on unravelling the mechanisms of O_2_ sensing in fish, there remain several significant unanswered questions. What are the molecular O_2_ sensors in O_2_ chemosensing cells? What is/are the neurotransmitter(s) secreted by O_2_ chemoreceptors? How are peripheral signals from the O_2_ chemoreceptors integrated within the CNS to evoke the HVR? Similar questions apply to the mechanisms of CO_2_ sensing, which have received much less attention. With respect to CO_2_ sensing, additional questions surround the nature of the critical stimulus that activates the CO_2_ sensor–is molecular CO_2_ itself sensed, or is the necessary stimulus a change in pH, and if so, is it extracellular or intracellular pH? A combination of CO_2_ and a resultant pH change is also possible. Further, for both O_2_ and CO_2_ sensing, there is a need to gather additional empirical evidence to demonstrate convincingly that NECs function as respiratory chemoreceptors *in vivo*. In our opinion, the absence of direct *in vivo* data to support the idea that NECs function as O_2_/CO_2_ chemoreceptors to regulate ventilation is THE limiting factor constraining progress in the field of piscine breathing control.

Providing the empirical evidence to answer these questions likely will be constrained by the technical challenges associated with identifying chemoreceptors *in vivo* or culturing chemoreceptor-nerve complexes *in vitro*. With recent advances in genetic manipulation, especially Tol2 based transgenesis and CRISPR/Cas9 based knockout (Zimmer et al., 2019), the zebrafish is emerging as an important model species to examine O_2_ and CO_2_ chemoreception *in vivo*. First and foremost, the promoters of *tph1a* and *vmat2* have been identified to be able to drive transgene expression in zebrafish skin and gill NECs (Pan et al., 2021a; Pan et al., 2021b; Pan et al., 2022). Given the transparency of zebrafish larvae and the ability to render adult zebrafish transparent by using the *Casper* line (White et al., 2008), a wide array of *in vivo* imaging techniques can now be applied. For example, calcium activity within NECs in response to changing levels of PO_2_ can be examined through *in vivo* calcium imaging using genetically encoded calcium indicators (Chen et al., 2013; Dana et al., 2016). Or, neurotransmitters being released by NECs under hypoxia can be examined by expressing specific neurotransmitter sensors (Wang et al., 2018) in NECs or the cranial sensory nerves that projects into the gill region (Kucenas et al., 2006).

An alternate approach that allows for experimental manipulation of sensory pathways is to express transgenes for cell activation, inhibition or ablation uniquely in NECs. Although genes such as *tph1a* and *vmat2* are expressed in NECs, they are also found in cells within the CNS that are critical for the regulation of breathing. Thus, driving transgenes with *tph1a* or *vmat2* promoters also would introduce transgene activity within the CNS, complicating data interpretation. It will be important, therefore, to identify genes that are expressed exclusively in NECs. Once achieved, NECs could be activated specifically with light or capsaicin by expressing channel rhodopsin (Antinucci et al., 2020) or rat TRPV1 channels (Matty et al., 2016). Similarly, NECs could be inhibited with light by expressing transient receptor potential cation channels (Antinucci et al., 2020) or ablated with metronidazole treatment in fish with NECs expressing nitroreductase (Sharrock et al., 2022). With such experiments, it would be possible to establish a direct link between NEC activity and ventilation. In summary, combining gene manipulation with physiological measurements offers a powerful approach to advance the field of chemoreception and control of breathing in fishes.

## References

[B1] Abdallah S. Jonz M. G. Perry S. F. (2015a). Extracellular H^+^ induces Ca^2+^ signals in respiratory chemoreceptors of zebrafish. Pflügers Arch. 467, 399–413. 10.1007/s00424-014-1514-2 24770973

[B2] Abdallah S. J. Thomas B. S. Jonz M. G. (2015b). Aquatic surface respiration and swimming behaviour in adult and developing zebrafish exposed to hypoxia. J. Exp. Biol. 218, 1777–1786. 10.1242/jeb.116343 25944921

[B3] Antinucci P. Dumitrescu A. Deleuze C. Morley H. J. Leung K. Hagley T. (2020). A calibrated optogenetic toolbox of stable zebrafish opsin lines. Elife 9, e54937. 10.7554/eLife.54937 32216873 PMC7170653

[B4] Bailly Y. Dunel-Erb S. Geffard M. Laurent P. (1989). The vascular and epithelial serotonergic innervation of the actinopterygian gill filament with special reference to the trout, *Salmo gairdneri* . Cell Tissue Res. 258, 349–363. 10.1007/bf00239455

[B5] Bailly Y. Dunel-Erb S. Laurent P. (1992). The neuroepithelial cells of the fish gill filament: Indolamine-immunocytochemistry and innervation. Anat. Rec. 233, 143–161. 10.1002/ar.1092330118 1605374

[B6] Bailly Y. (2009). “Serotonergic neuroepithelial cells in fish gills: Cytology and innervation,” in Airway chemoreceptors in the vertebrates. Editor Zaccone G. (Boca Raton: CRC Press), 61–98.

[B7] Bojink C. L. Florindo L. H. Leite C. a. C. Kalinin A. L. Milsom W. K. Rantin F. T. (2010). Hypercarbic cardiorespiratory reflexes in the facultative air-breathing fish jeju (*Hoplerythrinus unitaeniatus*): The role of branchial CO_2_ chemoreceptors. J. Exp. Biol. 213, 2797–2807. 10.1242/jeb.040733 20675550

[B8] Buckler K. J. (2012). Effects of exogenous hydrogen sulphide on calcium signalling, background (TASK) K channel activity and mitochondrial function in chemoreceptor cells. Pflügers Arch. 463, 743–754. 10.1007/s00424-012-1089-8 22419174 PMC3323823

[B9] Burggren W. W. (1982). Air gulping" improves blood oxygen transport during aquatic hypoxia in the goldfish *Carassius auratus* . Physiol. Zool. 55, 327–334. 10.1086/physzool.55.4.30155860

[B10] Burggren W. W. Bautista G. M. Coop S. C. Couturier G. M. Delgadillo S. P. García R. M. (2016). Developmental cardiorespiratory physiology of the air-breathing tropical gar, *Atractosteus tropicus* . Am. J. Physiol. 311, R689–R701. 10.1152/ajpregu.00022.2016 27465731

[B11] Burleson M. L. Mercer S. E. Wilk-Blaszczak M. A. (2006). Isolation and characterization of putative O_2_ chemoreceptor cells from the gills of channel catfish (*Ictalurus punctatus*). Brain Res. 1092, 100–107. 10.1016/j.brainres.2006.03.085 16690040

[B12] Burleson M. L. Milsom W. K. (1995). Cardio-ventilatory control in rainbow trout: I. Pharmacology of branchial, oxygen-sensitive chemoreceptors. Respir. Physiol. 100, 231–238. 10.1016/0034-5687(95)91595-x 7481112

[B13] Burleson M. L. Milsom W. K. (2003). “Comparative aspects of O_2_ chemoreception. Anatomy, physiology, and environmental applications,” in Oxygen sensing. Responses and adaptation to hypoxia. Editors Lahiri S. Semenza G. L. Prabhakar N. R. (New York: Marcel Dekker), 685–707.

[B14] Burleson M. L. Smatresk N. J. (2000). Branchial chemoreceptors mediate ventilatory responses to hypercapnic acidosis in channel catfish. Comp. Biochem. Physiol. 125A, 403–414. 10.1016/s1095-6433(00)00167-7 10794969

[B15] Burleson M. L. Smatresk N. J. Milsom W. K. (1992). “Afferent inputs associated with cardioventilatory control in fish,” in Fish Physiology v12B the cardiovascular system. Editors Hoar W. S. Randall D. J. Farrell A. P. (San Diego: Academic Press), 389–423.

[B16] Carroll J. L. (2003). Developmental plasticity in respiratory control. J. Appl. Physiol. 94, 375–389. 10.1152/japplphysiol.00809.2002 12486025

[B17] Chang A. J. Kim N. S. Hireed H. De Arce A. D. Ortega F. E. Riegler J. (2018). Chang et al. reply. Nature 561, E41. 10.1038/s41586-018-0547-7 30258152

[B18] Chang A. J. Ortega F. E. Riegler J. Madison D. V. Krasnow M. A. (2015). Oxygen regulation of breathing through an olfactory receptor activated by lactate. Nature 527, 240–244. 10.1038/nature15721 26560302 PMC4765808

[B19] Chapman L. J. McKenzie D. J. (2009). “Behavioral responses and ecological consequences,” in Fish Physiology v27 hypoxia. Editors Richards J. G. Farrell A. P. Brauner C. J. (USA: Elsevier), 25–77.

[B20] Chen T.-W. Wardill T. J. Sun Y. Pulver S. R. Renninger S. L. Baohan A. (2013). Ultrasensitive fluorescent proteins for imaging neuronal activity. Nature 499, 295–300. 10.1038/nature12354 23868258 PMC3777791

[B21] Coccimiglio M. L. Jonz M. G. (2012). Serotonergic neuroepithelial cells of the skin in developing zebrafish: Morphology, innervation and oxygen-sensitive properties. J. Exp. Biol. 215, 3881–3894. 10.1242/jeb.074575 22855620

[B22] Cochrane P. V. Jonz M. G. Wright P. A. (2021). The development of the O_2_-sensing system in an amphibious fish: Consequences of variation in environmental O_2_ levels. J. Comp. Physiol. B 191, 681–699. 10.1007/s00360-021-01379-5 34023926

[B23] Dana H. Mohar B. Sun Y. Narayan S. Gordus A. Hasseman J. P. (2016). Sensitive red protein calcium indicators for imaging neural activity. Elife 5, e12727. 10.7554/eLife.12727 27011354 PMC4846379

[B24] De Boeck G. Wood C. M. (2015). Does ammonia trigger hyperventilation in the elasmobranch, S*qualus acanthias suckleyi*? J. Exp. Biol. 206, 25–35. 10.1016/j.resp.2014.11.009 25462837

[B25] De Graaf P. Roberts B. (1991). Effects of vagal sensory input on the breathing rhythm of the carp. J. Exp. Biol. 155, 77–91. 10.1242/jeb.155.1.77

[B26] Diaz R. J. (2001). Overview of hypoxia around the world. J. Environ. Qual. 30, 275–281. 10.2134/jeq2001.302275x 11285887

[B27] Donnelly D. F. (2000). Developmental aspects of oxygen sensing by the carotid body. J. Appl. Physiol. 88, 2296–2301. 10.1152/jappl.2000.88.6.2296 10846048

[B28] Duchcherer M. Baghdadwala M. I. Paramonov J. Wilson R. J. (2013). Localization of essential rhombomeres for respiratory rhythm generation in bullfrog tadpoles using a binary search algorithm: Rhombomere 7 is essential for the gill rhythm and suppresses lung bursts before metamorphosis. Dev. Neurobiol. 73, 888–898. 10.1002/dneu.22108 23843256

[B29] Duchen M. Biscoe T. (1992a). Mitochondrial function in type I cells isolated from rabbit arterial chemoreceptors. J. Physiol. 450, 13–31. 10.1113/jphysiol.1992.sp019114 1432706 PMC1176109

[B30] Duchen M. Biscoe T. (1992b). Relative mitochondrial membrane potential and [Ca^2+^]_i_ in type I cells isolated from the rabbit carotid body. J. Physiol. 450, 33–61. 10.1113/jphysiol.1992.sp019115 1432712 PMC1176110

[B31] Dunel-Erb S. Bailly Y. Laurent P. (1982). Neuroepithelial cells in fish gill primary lamellae. J. Appl. Physiol. 53, 1342–1353. 10.1152/jappl.1982.53.6.1342 7153134

[B32] Eom J. Fehsenfeld S. Wood C. M. (2020). Is ammonia excretion affected by gill ventilation in the rainbow trout *Oncorhynchus mykiss*? Respir. Physiol. Neurobiol. 275, 103385. 10.1016/j.resp.2020.103385 31931176

[B33] Eom J. Giacomin M. Clifford A. M. Goss G. G. Wood C. M. (2019). Ventilatory sensitivity to ammonia in the Pacific hagfish (*Eptatretus stoutii*), a representative of the oldest extant connection to the ancestral vertebrates. J. Exp. Biol. 222, jeb199794. 10.1242/jeb.199794 31221739

[B34] Eom J. Wood C. M. (2021). Brain and gills as internal and external ammonia sensing organs for ventilatory control in rainbow trout, *Oncorhynchus mykiss* . Comp. Biochem. Physiol. A 254, 110896. 10.1016/j.cbpa.2021.110896 33444774

[B35] Evans A. M. (2004). “Hypoxia, cell metabolism, and cADPR accumulation,” in Hypoxic pulmonary vasoconstriction. Editor Yuan X. J. J., (New York: Springer), 313–338.

[B36] Fernandez-Aguera M. C. Gao L. Gonzalez-Rodriguez P. Pintado C. O. Arias-Mayenco I. Garcia-Flores P. (2015). Oxygen sensing by arterial chemoreceptors depends on mitochondrial complex I signaling. Cell. Metab. 22, 825–837. 10.1016/j.cmet.2015.09.004 26437605

[B37] Ferreira-Martins D. McCormick S. D. Campos A. Lopes-Marques M. Osório H. Coimbra J. (2016). A cytosolic carbonic anhydrase molecular switch occurs in the gills of metamorphic sea lamprey. Sci. Rep. 6, 33954. 10.1038/srep33954 27703170 PMC5050428

[B38] Florindo L. H. Reid S. G. Kalinin A. L. Milsom W. K. Rantin F. T. (2004). Cardiorespiratory reflexes and aquatic surface respiration in the neotropical fish tambaqui (*Colossoma macropomum*): Acute responses to hypercarbia. J. Comp. Physiol. B 174, 319–328. 10.1007/s00360-004-0417-5 14986045

[B39] Fu C. Wilson J. M. Rombough P. J. Brauner C. J. (2010). Ions first: Na^+^uptake shifts from the skin to the gills before O_2_ uptake in developing rainbow trout, *Oncorhynchus mykiss* . Proc. R. Soc. Lond. B 277, 1553–1560. 10.1098/rspb.2009.1545 PMC2871827 20071386

[B40] Furch K. Junk W. J. (1997). “Physicochemical conditions in the floodplains,” in The central Amazon floodplain. Editor Junk W. J. (Berlin, Heidelberg: Springer), 69–108.

[B41] Gao L. González-Rodríguez P. Ortega-Sáenz P. López-Barneo J. (2017). Redox signaling in acute oxygen sensing. Redox Biol. 12, 908–915. 10.1016/j.redox.2017.04.033 28476010 PMC5426049

[B42] Gee J. H. Tallman R. F. Smart H. J. (1978). Reactions of some great plains fishes to progressive hypoxia. Can. J. Zool. 56, 1962–1966. 10.1139/z78-263

[B43] Gilmour K. M. Milsom W. K. Rantin F. T. Reid S. G. Perry S. F. (2005). Cardiorespiratory responses to hypercarbia in tambaqui (*Colossoma macropomum*): Chemoreceptor orientation and specificity. J. Exp. Biol. 208, 1095–1107. 10.1242/jeb.01480 15767310

[B44] Gilmour K. M. Perry S. F. (2007). “Branchial chemoreceptor regulation of cardiorespiratory function,” in Fish Physiology v25 sensory systems neuroscience. Editors Hara T. J. Zielinski B. (USA: Elsevier), 97–151.

[B45] Gilmour K. M. (2001). The CO_2_/pH ventilatory drive in fish. Comp. Biochem. Physiol. 130A, 219–240. 10.1016/s1095-6433(01)00391-9 11544069

[B46] Glass M. L. (1992). “Ventilatory responses to hypoxia in ectothermic vertebrates,” in Physiological adaptations in vertebrates, respiration, circulation, and metabolism. Editors Wood S. C. Weber R. E. Hargens A. R. Millard R. W. (New York: Marcel Dekker), 97–118.

[B47] Gonzalez C. Agapito M. T. Rocher A. Gomez-Nino A. Rigual R. Castaneda J. (2010). A revisit to O_2_ sensing and transduction in the carotid body chemoreceptors in the context of reactive oxygen species biology. Respir. Physiol. Neurobiol. 174, 317–330. 10.1016/j.resp.2010.09.002 20833275

[B48] Heisler N. Toews D. P. Holeton G. F. (1988). Regulation of ventilation and acid-base status in the elasmobranch *Scyliorhinus stellaris* during hyperoxia induced hypercapnia. Respir. Physiol. 71, 227–246. 10.1016/0034-5687(88)90018-7 3124238

[B49] Ho D. H. Burggren W. W. (2012). Parental hypoxic exposure confers offspring hypoxia resistance in zebrafish (*Danio rerio*). J. Exp. Biol. 215, 4208–4216. 10.1242/jeb.074781 22899535

[B50] Holeton G. F. Randall D. J. (1967). The effect of hypoxia upon the partial pressure of gases in the blood and water afferent and efferent to the gills of rainbow trout. J. Exp. Biol. 46, 317–327. 10.1242/jeb.46.2.317 6033998

[B51] Holeton G. F. (1971). Respiratory and circulatory responses of rainbow trout larvae to carbon monoxide and to hypoxia. J. Exp. Biol. 55, 683–694. 10.1242/jeb.55.3.683 5160862

[B52] Iwama G. K. Boutilier R. G. Heming T. A. Randall D. J. Mazeaud M. (1987). The effects of altering gill water flow on gas transfer in rainbow trout. Can. J. Zool. 65, 2466–2470. 10.1139/z87-372

[B53] Jones D. R. Schwarzfeld T. (1974). The oxygen cost to the metabolism and efficiency of breathing in trout (*Salmo gairdneri*). Respir. Physiol. 21, 241–254. 10.1016/0034-5687(74)90097-8 4416396

[B54] Jonz M. G. Buck L. T. Perry S. F. Schwerte T. Zaccone G. (2015a). Sensing and surviving hypoxia in vertebrates. Ann. N.Y. Acad. Sci. 1365, 43–58. 10.1111/nyas.12780 25959851

[B55] Jonz M. G. Fearon I. M. Nurse C. A. (2004). Neuroepithelial oxygen chemoreceptors of the zebrafish gill. J. Physiol. 560, 737–752. 10.1113/jphysiol.2004.069294 15331683 PMC1665292

[B56] Jonz M. G. Nurse C. A. (2005). Development of oxygen sensing in the gills of zebrafish. J. Exp. Biol. 208, 1537–1549. 10.1242/jeb.01564 15802677

[B57] Jonz M. G. Nurse C. A. (2003). Neuroepithelial cells and associated innervation of the zebrafish gill: A confocal immunofluorescence study. J. Comp. Neurol. 461, 1–17. 10.1002/cne.10680 12722101

[B58] Jonz M. G. Nurse C. A. (2006). Ontogenesis of oxygen chemoreception in aquatic vertebrates. Respir. Physiol. Neurobiol. 154, 139–152. 10.1016/j.resp.2006.01.004 16488670

[B59] Jonz M. G. Zachar P. C. Da Fonte D. F. Mierzwa A. S. (2015b). Peripheral chemoreceptors in fish: A brief history and a look ahead. Comp. Biochem. Physiol. A 186, 27–38. 10.1016/j.cbpa.2014.09.002 25218943

[B60] Kinkead R. Perry S. F. (1991). The effects of catecholamines on ventilation in rainbow trout during hypoxia or hypercapnia. Respir. Physiol. 84, 77–92. 10.1016/0034-5687(91)90020-j 1906629

[B61] Koudrina N. Perry S. F. Gilmour K. M. (2020). The role of TASK-2 channels in CO_2_ sensing in zebrafish (*Danio rerio*). Am. J. Physiol. 319, R329–R342. 10.1152/ajpregu.00132.2020 PMC7509255 32697653

[B62] Kramer D. L. McClure M. (1982). Aquatic surface respiration, a widespread adaptation to hypoxia in tropical freshwater fishes. Env. Biol. Fish. 7, 47–55. 10.1007/bf00011822

[B63] Kramer D. L. Mehegan J. P. (1981). Aquatic surface respiration, an adaptive response to hypoxia in the guppy, *Poecilia reticulata* (Pisces, Poeciliidae). Env. Biol. Fish. 6, 299–313. 10.1007/bf00005759

[B64] Kucenas S. Soto F. Cox J. Voigt M. (2006). Selective labeling of central and peripheral sensory neurons in the developing zebrafish using P2X3 receptor subunit transgenes. Neurosci 138, 641–652. 10.1016/j.neuroscience.2005.11.058 16413125

[B65] Kumar P. Bin-Jaliah I. (2007). Adequate stimuli of the carotid body: More than an oxygen sensor? Respir. Physiol. Neurobiol. 157, 12–21. 10.1016/j.resp.2007.01.007 17291838

[B66] Kunert E. Joyce W. Pan Y. K. Chen A. Perry S. F. Gilmour K. M. (2022). The role of cytosolic carbonic anhydrase Ca17a in cardiorespiratory responses to CO_2_ in developing zebrafish (*Danio rerio*). Am. J. Physiol. 323, R532–R546. 10.1152/ajpregu.00050.2022 35993559

[B67] Leonard E. M. Salman S. Nurse C. A. (2018). Sensory processing and integration at the carotid body tripartite synapse: Neurotransmitter functions and effects of chronic hypoxia. Front. Physiol. 9, 225. 10.3389/fphys.2018.00225 29615922 PMC5864924

[B68] Leonard E. M. Weaver F. E. Nurse C. A. (2022). Lactate sensing by neuroepithelial cells isolated from the gills of killifish (*Fundulus heteroclitus*). J. Exp. Biol. 225, jeb245088. 10.1242/jeb.245088 36420741

[B69] Li Q. Sun B. Wang X. Jin Z. Zhou Y. Dong L. (2010). A crucial role for hydrogen sulfide in oxygen sensing via modulating large conductance calcium-activated potassium channels. Antioxid. Redox Signal. 12, 1179–1189. 10.1089/ars.2009.2926 19803741

[B70] Lindsey B. W. Smith F. M. Croll R. P. (2010). From inflation to flotation: Contribution of the swimbladder to whole-body density and swimming depth during development of the zebrafish (*Danio rerio*). Zebrafish 7, 85–96. 10.1089/zeb.2009.0616 20415646

[B71] Ling L. Olson E. B. Vidruk E. H. Mitchell G. S. (1997). Developmental plasticity of the hypoxic ventilatory response. Respir. Physiol. 110, 261–268. 10.1016/s0034-5687(97)00091-1 9407619

[B72] Lopez-Barneo J. Gonzalez-Rodriguez P. Gao L. Fernandez-Aguera M. C. Pardal R. Ortega-Saenz P. (2016). Oxygen sensing by the carotid body: Mechanisms and role in adaptation to hypoxia. Am. J. Physiol. 310, C629–C642. 10.1152/ajpcell.00265.2015 26764048

[B73] Mandic M. Flear K. Qiu P. Pan Y. K. Perry S. F. Gilmour K. M. (2022). Aquatic surface respiration significantly improves survival of zebrafish (*Danio rerio*) lacking hypoxia inducible factor 1-α. Proc. R. Soc. Lond. B 289, 20211863. 10.1098/rspb.2021.1863 PMC8753152 35016541

[B74] Mandic M. Pan Y. K. Gilmour K. M. Perry S. F. (2020). Relationships between the peak hypoxic ventilatory response and critical O_2_ tension in larval and adult zebrafish (*Danio rerio*). J. Exp. Biol. 223, jeb213942. jeb.213942. 10.1242/jeb.213942 32139474

[B75] Mandic M. Tzaneva V. Careau V. Perry S. F. (2019). Hif-1α paralogs play a role in the hypoxic ventilatory response of larval and adult zebrafish (*Danio rerio*). J. Exp. Biol. 222, jeb195198. 10.1242/jeb.195198 30518608

[B76] Matty M. A. Beerman R. W. Tobin D. M. (2016). Drug-inducible, cell-specific manipulation of intracellular calcium in Zebrafish through mammalian TRPV1 expression. Zebrafish 13, 374–375. 10.1089/zeb.2016.29004.mat 27058231 PMC4994051

[B77] McDonald D. G. McMahon B. R. (1977). Respiratory development in Arctic char *Salvelinus alpinus* under conditions of normoxia and chronic hypoxia. Can. J. Zool. 55, 1461–1467. 10.1139/z77-189 907929

[B78] McKendry J. E. Milsom W. K. Perry S. F. (2001). Branchial CO_2_ receptors and cardiorespiratory adjustments during hypercarbia in Pacific spiny dogfish (*Squalus acanthias*). J. Exp. Biol. 204, 1519–1527. 10.1242/jeb.204.8.1519 11273813

[B79] Mellen N. M. Thoby-Brisson M. (2012). Respiratory circuits: Development, function and models. Curr. Opin. Neurobiol. 22, 676–685. 10.1016/j.conb.2012.01.001 22281058

[B80] Miller S. Pollack J. Bradshaw J. Kumai Y. Perry S. F. (2014). Cardiac responses to hypercapnia in larval zebrafish (*Danio rerio*): The links between CO_2_ chemoreception, catecholamines and carbonic anhydrase. J. Exp. Biol. 217, 3569–3578. 10.1242/jeb.107987 25063853

[B81] Milsom W. K. Gilmour K. M. Perry S. F. Gargaglioni L. Hedrick M. Kinkead R. (2022). Control of breathing in ectothermic vertebrates. Compr. Physiol. 12, 3869–3988. 10.1002/cphy.c210041 35997081

[B82] Milsom W. K. (2012). New insights into gill chemoreception: Receptor distribution and roles in water and air breathing fish. Respir. Physiol. Neurobiol. 184, 326–339. 10.1016/j.resp.2012.07.013 22841952

[B83] Milsom W. K. Reid S. G. Rantin F. T. Sundin L. (2002). Extrabranchial chemoreceptors involved in respiratory reflexes in the neotropical fish *Colossoma macropomum* (the tambaqui). J. Exp. Biol. 205, 1765–1774. 10.1242/jeb.205.12.1765 12042335

[B84] Olson K. R. Donald J. A. Dombkowski R. A. Perry S. F. (2012). Evolutionary and comparative aspects of nitric oxide, carbon monoxide and hydrogen sulfide. Respir. Physiol. Neurobiol. 184, 117–129. 10.1016/j.resp.2012.04.004 22546339

[B85] Olson K. R. Healy M. Zhaohong Q. Vulesevic B. Duff D. W. Whitfield N. (2008). Hydrogen sulfide as an oxygen sensor in trout gill chemoreceptors. Am. J. Physiol. 295, R669–R680. 10.1152/ajpregu.00807.2007 18565835

[B86] Ortega-Sáenz P. Moreno-Domínguez A. Gao L. López-Barneo J. (2020). Molecular mechanisms of acute oxygen sensing by arterial chemoreceptor cells. Role of Hif2α. Front. Physiol. 11, 614893. 10.3389/fphys.2020.614893 33329066 PMC7719705

[B87] Pan W. Godoy R. S. Cook D. P. Scott A. L. Nurse C. A. Jonz M. G. (2022). Single-cell transcriptomic analysis of neuroepithelial cells and other cell types of the gills of zebrafish (*Danio rerio*) exposed to hypoxia. Sci. Rep. 12, 10144. 10.1038/s41598-022-13693-1 35710785 PMC9203529

[B88] Pan W. Scott A. L. Nurse C. A. Jonz M. G. (2021a). Identification of oxygen-sensitive neuroepithelial cells through an endogenous reporter gene in larval and adult transgenic zebrafish. Cell Tissue Res. 384, 35–47. 10.1007/s00441-020-03307-5 33404838

[B89] Pan Y. K. Jensen G. Perry S. F. (2021b). Disruption of tph1 genes demonstrates the importance of serotonin in regulating ventilation in larval zebrafish (*Danio rerio*). Respir. Physiol. Neurobiol. 285, 103594. 10.1016/j.resp.2020.103594 33271304

[B90] Pan Y. K. Mandic M. Zimmer A. M. Perry S. F. (2019). Evaluating the physiological significance of hypoxic hyperventilation in larval zebrafish (*Danio rerio*). J. Exp. Biol. 222, jeb204800. 10.1242/jeb.204800 31196977

[B91] Pan Y. K. Perry S. F. (2020). Neuroendocrine control of breathing in fish. Mol. Cell. Endocrinol. 509, 110800. 10.1016/j.mce.2020.110800 32240728

[B92] Peña-Münzenmayer G. Niemeyer M. I. Sepúlveda F. V. Cid L. P. (2013). Zebrafish and mouse TASK-2 K^+^ channels are inhibited by increased CO_2_ and intracellular acidification. Pflügers Arch. 466, 1317–1327. 10.1007/s00424-013-1365-2 24081451

[B93] Perry S. F. Abdallah S. J. (2012). Mechanisms and consequences of carbon dioxide sensing in fish. Respir. Physiol. Neurobiol. 184, 309–315. 10.1016/j.resp.2012.06.013 22705499

[B94] Perry S. F. Esbaugh A. Braun M. Gilmour K. M. (2009a). “Gas transport and gill function in water-breathing fish,” in Cardio-respiratory control in vertebrates: Comparative and evolutionary aspects. Editors Glass M. L. Wood S. C. (Heidelberg: Springer), 5–42.

[B95] Perry S. F. Gilmour K. M. (2010). “Oxygen uptake and transport in water breathers,” in Respiratory Physiology of vertebrates: Life with and without oxygen. Editor Nilsson G. E. (Cambridge: Cambridge University Press), 49–94.

[B96] Perry S. F. Gilmour K. M. (2002). Sensing and transfer of respiratory gases at the fish gill. J. Exp. Zool. 293, 249–263. 10.1002/jez.10129 12115900

[B97] Perry S. F. Jonz M. G. Gilmour K. M. (2009b). “Oxygen sensing and the hypoxic ventilatory response,” in Fish Physiology v27 hypoxia. Editors Richards J. G. Brauner C. J. Farrell A. P. (Amsterdam: Academic Press), 193–251.

[B98] Perry S. F. Mckendry J. E. (2001). The relative roles of external and internal CO_2_ *versus* H^+^ in eliciting the cardiorespiratory responses of *Salmo salar* and *Squalus acanthias* to hpercarbia. J. Exp. Biol. 204, 3963–3971. 10.1242/jeb.204.22.3963 11807114

[B99] Perry S. F. Reid S. G. (2002). Cardiorespiratory adjustments during hypercarbia in rainbow trout *Oncorhynchus mykiss* are initiated by external CO_2_ receptors on the first gill arch. J. Exp. Biol. 205, 3357–3365. 10.1242/jeb.205.21.3357 12324545

[B100] Perry S. F. (2011). “Respiratory responses to hypoxia in fishes,” in Encyclopaedia of fish Physiology: From genome to environment. Editor Farrell A. P. (San Diego: Elsevier), 1751–1756.

[B101] Perry S. F. Tzaneva V. (2016). The sensing of respiratory gases in fish: Mechanisms and signalling pathways. Respir. Physiol. Neurobiol. 224, 71–79. 10.1016/j.resp.2015.06.007 26130330

[B102] Perry S. Kumai Y. Porteus C. Tzaneva V. Kwong R. (2016). An emerging role for gasotransmitters in the control of breathing and ionic regulation in fish. J. Comp. Physiol. B 186, 145–159. 10.1007/s00360-015-0949-x 26660653

[B103] Polymeropoulos E. T. Plouffe D. Leblanc S. Elliott N. G. Currie S. Frappell P. B. (2014). Growth hormone transgenesis and polyploidy increase metabolic rate, alter the cardiorespiratory response and influence HSP expression in response to acute hypoxia in Atlantic salmon (*Salmo salar*) yolk-sac alevins. J. Exp. Biol. 217, 2268–2276. 10.1242/jeb.098913 24675560

[B104] Porteus C. Abdallah S. J. Pollack J. Kumai Y. Kwong R. W. M. Yew H. M. (2014). The role of hydrogen sulphide in the control of breathing in hypoxic zebrafish (*Danio rerio*). J. Physiol. 592, 3075–3088. 10.1113/jphysiol.2014.271098 24756639 PMC4214661

[B105] Porteus C. Kumai Y. Abdallah S. J. Yew H. M. Kwong R. W. M. Pan Y. K. (2021). Respiratory responses to external ammonia in zebrafish (*Danio rerio*). Comp. Biochem. Physiol. A 251, 110822. 10.1016/j.cbpa.2020.110822 33059022

[B106] Porteus C. S. Brink D. L. Milsom W. K. (2012). Neurotransmitter profiles in fish gills: Putative gill oxygen chemoreceptors. Respir. Physiol. Neurobiol. 184, 316–325. 10.1016/j.resp.2012.06.019 22728948

[B107] Porteus C. S. Pollack J. Tzaneva V. Kwong R. W. M. Kumai Y. Abdallah S. J. (2015). A role for nitric oxide in the control of breathing in zebrafish (*Danio rerio*). J. Exp. Biol. 218, 3746–3753. 10.1242/jeb.127795 26486367

[B108] Prabhakar N. R. (2013). Sensing hypoxia: Physiology, genetics and epigenetics. J. Physiol. 591, 2245–2257. 10.1113/jphysiol.2012.247759 23459758 PMC3650692

[B109] Qin Z. Lewis J. E. Perry S. F. (2010). Zebrafish (*Danio rerio*) gill neuroepithelial cells are sensitive chemoreceptors for environmental CO_2_ . J. Physiol. 588, 861–872. 10.1113/jphysiol.2009.184739 20051495 PMC2834944

[B110] Ragsdale A. Ortega-Recalde O. Dutoit L. Besson A. A. Chia J. H. Z. King T. (2022). Paternal hypoxia exposure primes offspring for increased hypoxia resistance. BMC Biol. 20, 185. 10.1186/s12915-022-01389-x 36038899 PMC9426223

[B111] Rakoczy R. J. Wyatt C. N. (2018). Acute oxygen sensing by the carotid body: A rattlebag of molecular mechanisms. J. Physiol. 596, 2969–2976. 10.1113/JP274351 29214644 PMC6068253

[B112] Randall D. J. Ip Y. K. (2006). Ammonia as a respiratory gas in water and air-breathing fishes. Respir. Physiol. Neurobiol. 154, 216–225. 10.1016/j.resp.2006.04.003 16731054

[B113] Randall D. J. Shelton G. (1963). The effects of changes in environmental gas concentrations on the breathing and heart rate of a teleost fish. Comp. Biochem. Physiol. 9, 229–239. 10.1016/0010-406x(63)90046-x 14111889

[B114] Randall D. J. (1982). The control of respiration and circulation in fish during exercise and hypoxia. J. Exp. Biol. 100, 275–288. 10.1242/jeb.100.1.275

[B115] Rasera M. D. F. L. Krusche A. V. Richey J. E. Ballester M. V. R. Victória R. L. (2013). Spatial and temporal variability of pCO_2_ and CO_2_ efflux in seven Amazonian Rivers. Biogeochem 116, 241–259. 10.1007/s10533-013-9854-0

[B116] Reed M. Jonz M. G. (2022). Neurochemical signalling associated with gill oxygen sensing and ventilation: A receptor focused mini-review. Front. Physiol. 13, 940020. 10.3389/fphys.2022.940020 35910553 PMC9325958

[B117] Regan K. S. Jonz M. G. Wright P. A. (2011). Neuroepithelial cells and the hypoxia emersion response in the amphibious fish *Kryptolebias marmoratus* . J. Exp. Biol. 214, 2560–2568. 10.1242/jeb.056333 21753050

[B118] Reid S. G. Perry S. F. Gilmour K. M. Milsom W. K. Rantin F. T. (2005). Reciprocal modulation of O_2_ and CO_2_ cardiorespiratory chemoreflexes in the tambaqui. Respir. Physiol. Neurobiol. 146, 175–194. 10.1016/j.resp.2004.12.008 15766906

[B119] Reid S. G. Sundin L. Kalinin A. L. Rantin F. T. Milsom W. K. (2000). Cardiovascular and respiratory reflexes in the tropical fish, traira (*Hoplias malabaricus*): CO_2_/pH chemoresponses. Respir. Physiol. 120, 47–59. 10.1016/s0034-5687(99)00100-0 10786644

[B120] Robertson C. E. Turko A. J. Jonz M. G. Wright P. A. (2015). Hypercapnia and low pH induce neuroepithelial cell proliferation and emersion behaviour in the amphibious fish *Kryptolebias marmoratus* . J. Exp. Biol. 218, 2987–2990. 10.1242/jeb.123133 26254321

[B121] Rombough P. (2002). Gills are needed for ionoregulation before they are needed for O_2_ uptake in developing zebrafish, *Danio rerio* . J. Exp. Biol. 205, 1787–1794. 10.1242/jeb.205.12.1787 12042337

[B122] Rombough P. J. (1988). “Respiratory gas exchange, aerobic metabolism, and effects of hypoxia during early life,” in Fish Physiology vXIA. Editors Hoar W. S. Randall D. J. (San Diego: Academic Press), 59–161.

[B123] Rombough P. (2007). The functional ontogeny of the teleost gill: Which comes first, gas or ion exchange? Comp. Biochem. Physiol. A 148, 732–742. 10.1016/j.cbpa.2007.03.007 17451987

[B124] Rosales K. Yeh C.-M. How J. J. Villa-Real R. Depasquale E. Groisman A. (2019). Neural pathways linking hypoxia with pectoral fin movements in *Danio rerio* . bioRxiv, 10.1101/655084

[B125] Rossi G. S. Cochrane P. V. Wright P. A. (2020). Fluctuating environments during early development can limit adult phenotypic flexibility: Insights from an amphibious fish. J. Exp. Biol. 223, jeb228304. 10.1242/jeb.228304 32616545

[B126] Shakarchi K. Zachar P. C. Jonz M. G. (2013). Serotonergic and cholinergic elements of the hypoxic ventilatory response in developing zebrafish. J. Exp. Biol. 216, 869–880. 10.1242/jeb.079657 23155078

[B127] Sharrock A. V. Mulligan T. S. Hall K. R. Williams E. M. White D. T. Zhang L. (2022). Ntr 2.0: A rationally engineered prodrug-converting enzyme with substantially enhanced efficacy for targeted cell ablation. Nat. Methods 19, 205–215. 10.1038/s41592-021-01364-4 35132245 PMC8851868

[B128] Shelton G. Jones D. R. Milsom W. K. (1986). “Control of breathing in ectothermic vertebrates,” in Handbook of Physiology, section 3. The respiratory system. Editors Cherniak N. S. Widdicombe J. G. (Bethesda: American Physiological Society), 2, 857–909. Control of Breathing.

[B129] Sloman K. A. Mandic M. Todgham A. E. Fangue N. A. Subrt P. Richards J. G. (2008). The response of the tidepool sculpin, *Oligocottus maculosus*, to hypoxia in laboratory, mesocosm and field environments. Comp. Biochem. Physiol. A 149, 284–292. 10.1016/j.cbpa.2008.01.004 18276177

[B130] Steffensen J. F. Lomholt J. P. (1983). Energetic cost of active branchial ventilation in the sharksucker, *Echeneis naucrates* . J. Exp. Biol. 103, 185–192. 10.1242/jeb.103.1.185 6854201

[B131] Sundin L. Burleson M. L. Sanchez A. P. Amin-Naves J. Kinkead R. Gargaglioni L. H. (2007). Respiratory chemoreceptor function in vertebrates comparative and evolutionary aspects. Integr. Comp. Biol. 47, 592–600. 10.1093/icb/icm076 21672865

[B132] Sundin L. Reid S. G. Rantin F. T. Milsom W. K. (2000). Branchial receptors and cardiorespiratory reflexes in the neotropical fish, Tambaqui (*Colossoma macropomum*). J. Exp. Biol. 203, 1225–1239. 10.1242/jeb.203.7.1225 10708642

[B133] Thomsen M. T. Lefevre S. Nilsson G. E. Wang T. Bayley M. (2019). Effects of lactate ions on the cardiorespiratory system in rainbow trout (*Oncorhynchus mykiss*). Am. J. Physiol. 316, R607–R620. 10.1152/ajpregu.00395.2018 30811217

[B134] Thomsen M. T. Wang T. Milsom W. K. Bayley M. (2017). Lactate provides a strong pH-independent ventilatory signal in the facultative air-breathing teleost *Pangasianodon hypophthalmus* . Sci. Rep. 7, 6378–6379. 10.1038/s41598-017-06745-4 28743938 PMC5527003

[B135] Torres-Torrelo H. Ortega-Sáenz P. Macías D. Omura M. Zhou T. Matsunami H. (2018). The role of Olfr78 in the breathing circuit of mice. Nature 561, E33–E40. 10.1038/s41586-018-0545-9 30258151

[B136] Tresguerres M. Milsom W. K. Perry S. F. (2019). “CO_2_ and acid-base sensing,” in Fish Physiology v37 carbon dioxide. Editors Grosell M. Munday P. L. Farrell A. P. Brauner C. J. (USA: Elsevier), 33–68.

[B137] Turesson J. Schwerte T. Sundin L. (2006). Late onset of NMDA receptor-mediated ventilatory control during early development in zebrafish (*Danio rerio*). Comp. Biochem. Physiol. A 143, 332–339. 10.1016/j.cbpa.2005.12.008 16458555

[B138] Tzaneva V. Perry S. F. (2014). Heme oxygenase-1 (HO-1) mediated respiratory responses in the goldfish, *Carassius auratus* . Resp. Physiol. Neurobiol. 199, 1–8. 10.1016/j.resp.2014.04.006 24780551

[B139] Tzaneva V. Perry S. F. (2016). Role of endogenous carbon monoxide in the control of breathing in zebrafish (*Danio rerio*). Am. J. Physiol. 311, R1262–R1270. 10.1152/ajpregu.00094.2016 PMC5256984 27581810

[B140] Varas R. Wyatt C. N. Buckler K. J. (2007). Modulation of TASK‐like background potassium channels in rat arterial chemoreceptor cells by intracellular ATP and other nucleotides. J. Physiol. 583, 521–536. 10.1113/jphysiol.2007.135657 17615104 PMC2156202

[B141] Vulesevic B. McNeill B. Perry S. F. (2006). Chemoreceptor plasticity and respiratory acclimation in the zebrafish, *Danio rerio* . J. Exp. Biol. 209, 1261–1273. 10.1242/jeb.02058 16547298

[B142] Vulesevic B. Perry S. F. (2006). Developmental plasticity of ventilatory control in zebrafish, *Danio rerio* . Respir. Physiol. Neurobiol. 154, 396–405. 10.1016/j.resp.2006.01.001 16446127

[B143] Wang H. Jing M. Li Y. (2018). Lighting up the brain: Genetically encoded fluorescent sensors for imaging neurotransmitters and neuromodulators. Curr. Opin. Neurobiol. 50, 171–178. 10.1016/j.conb.2018.03.010 29627516 PMC5984720

[B144] Weber J.-M. Kramer D. L. (1983). Effects of hypoxia and surface access on growth, mortality, and behavior of juvenile guppies, *Poecilia reticulata* . Can. J. Fish. Aquat. Sci. 40, 1583–1588. 10.1139/f83-183

[B145] White R. M. Sessa A. Burke C. Bowman T. Leblanc J. Ceol C. (2008). Transparent adult zebrafish as a tool for *in vivo* transplantation analysis. Cell Stem Cell 2, 183–189. 10.1016/j.stem.2007.11.002 18371439 PMC2292119

[B146] Wood C. M. Jackson E. B. (1980). Blood acid-base regulation during environmental hyperoxia in the rainbow trout (*Salmo gairdneri*). Respir. Physiol. 42, 351–372. 10.1016/0034-5687(80)90125-5 6784212

[B147] Wood C. M. Munger R. S. (1994). Carbonic anhydrase injection provides evidence for the role of blood acid-base status in stimulating ventilation after exhaustive exercise in rainbow trout. J. Exp. Biol. 194, 225–253. 10.1242/jeb.194.1.225 9317687

[B148] Wyatt C. N. Buckler K. J. (2004). The effect of mitochondrial inhibitors on membrane currents in isolated neonatal rat carotid body type I cells. J. Physiol. 556, 175–191. 10.1113/jphysiol.2003.058131 14724184 PMC1664886

[B149] Yuan G. Vasavda C. Peng Y.-J. Makarenko V. V. Raghuraman G. Nanduri J. (2015). Protein kinase G–regulated production of H_2_S governs oxygen sensing. Sci. Signal. 8, ra37. 10.1126/scisignal.2005846 25900831 PMC4418480

[B150] Zaccone G. Lauriano E. R. Kuciel M. Capillo G. Pergolizzi S. Alesci A. (2017). Identification and distribution of neuronal nitric oxide synthase and neurochemical markers in the neuroepithelial cells of the gill and the skin in the giant mudskipper, *Periophthalmodon schlosseri* . Periophthalmodon Schlosseri. Zool. 125, 41–52. 10.1016/j.zool.2017.08.002 28830730

[B151] Zachar P. C. Jonz M. G. (2012). Neuroepithelial cells of the gill and their role in oxygen sensing. Respir. Physiol. Neurobiol. 184, 301–308. 10.1016/j.resp.2012.06.024 22772312

[B152] Zachar P. C. Pan W. Jonz M. G. (2017). Characterization of ion channels and O_2_ sensitivity in gill neuroepithelial cells of the anoxia-tolerant goldfish (*Carassius auratus*). J. Neurophysiol. 118, 3014–3023. 10.1152/jn.00237.2017 28904098 PMC5712667

[B153] Zhang L. Nawata C. M. Wood C. M. (2013). Sensitivity of ventilation and brain metabolism to ammonia exposure in rainbow trout, *Oncorhynchus mykiss* . J. Exp. Biol. 216, 4025–4037. 10.1242/jeb.087692 23868844

[B154] Zhang L. Nawata M. De Boeck G. Wood C. M. (2015). Rh protein expression in branchial neuroepithelial cells, and the role of ammonia in ventilatory control in fish. Comp. Biochem. Physiol. 186A, 39–51. 10.1016/j.cbpa.2014.10.004 25465530

[B155] Zhang L. Nurse C. A. Jonz M. G. Wood C. M. (2011). Ammonia sensing by neuroepithelial cells and ventilatory responses to ammonia in rainbow trout. J. Exp. Biol. 214, 2678–2689. 10.1242/jeb.055541 21795563

[B156] Zhang L. Wood C. M. (2009). Ammonia as a stimulant to ventilation in rainbow trout *Oncorhynchus mykiss* . Respir. Physiol. Neurobiol. 168, 261–271. 10.1016/j.resp.2009.07.011 19619676

[B157] Zimmer A. M. Mandic M. Rourke K. M. Perry S. F. (2020). Breathing with fins: Do the pectoral fins of larval fishes play a respiratory role? Am. J. Physiol. 318, R89–R97. 10.1152/ajpregu.00265.2019 31692366

[B158] Zimmer A. M. Pan Y. K. Chandrapalan T. Kwong R. W. M. Perry S. F. (2019). Loss-of-function approaches in comparative physiology: Is there a future for knockdown experiments in the era of genome editing? J. Exp. Biol. 222, jeb175737. 10.1242/jeb.175737 30948498

[B159] Zimmer A. M. Perry S. F. (2022). Physiology and aquaculture: A review of ion and acid‐base regulation by the gills of fishes. Fish. Fish. 23, 874–898. 10.1111/faf.12659

